# Divergent response associates with the differential amplitudes of immunity against *Magnaporthe oryzae* by different blast resistance genes

**DOI:** 10.3389/fpls.2025.1547593

**Published:** 2025-02-24

**Authors:** Beenish Hassan, Sadam Hussain Bhutto, Xiao-Xiao Yin, Xiu-Lian Yan, Rong Liao, Mao-Lin Guo, Ya-Ping Tang, Dai-Ming Guo, Si-Jia Yang, Faiza Gulzar, Yan Li, Xian-Yin Zeng, Zhi-Xue Zhao, Wen-Ming Wang

**Affiliations:** ^1^ State Key Laboratory of Crop Gene Exploration and Utilization in Southwest China, Sichuan Agricultural University, Chengdu, China; ^2^ Rice Research Institute, Sichuan Agricultural University, Chengdu, China; ^3^ Department of Engineering and Applied Biology, College of Life Science, Sichuan Agricultural University, Ya’an, China

**Keywords:** rice, *Magnaporthe oryzae*, rice blast disease, blast resistance genes, immune response

## Abstract

Rice blast disease, caused by *Magnaporthe oryzae*, poses the most devastating threat to global rice production. The products of most blast resistance (*R*) genes specifically recognize corresponding a virulence effectors from the pathogen, thereby mediating robust immune responses that are crucial for disease resistance. However, it is unclear why different *R* genes endow with differential amplitudes of immunity against *M*. *oryzae*. Here, we demonstrated that different blast *R* genes confer differential amplitudes of immunity against *M*. *oryzae*, presumably due to divergent reprogramming of transcriptional responses. We detected that three rice restorer lines exhibited differential amplitudes of immune responses, despite all lines displaying resistance to *M*. *oryzae*. Consistently, different accessions carrying different single *R* genes exhibited remarkable differentially expressed genes (DEGs) count, indicating different transcriptional re-programming that leads to different fitness cost. Comparative analysis revealed varying degrees of overlap among DEGs across different accessions. By integrating RNA-seq and RT-qPCR data, we recommended some marker genes that distinguish the differential amplitude of immunity against *M*. *oryzae* mediated by different blast *R* genes. Thus, our study provides valuable insights into the specific and overlapping roles of *R* gene-mediated immunity. We also propose marker genes that can be used to effectively evaluate the amplitude of immune responses to *M*. *oryzae*, thereby facilitating the assessment of *R* genes with relatively lower amplitude of immunity in order to minimize fitness cost.

## Introduction

Rice has evolved a two-layered immune system to defend against blast fungal invasion. The first layer, known as pathogen-associated molecular patterns (PAMPs)-triggered immunity (PTI) (Saile et al., 2020), is activated upon the recognition of PAMPs by pattern recognition receptors (PRRs) (Jones and Dangl, 2006; Couto and Zipfel, 2016; Yu et al., 2017). The second layer, termed effector-triggered immunity (ETI), is triggered upon the detection of effectors by blast resistance genes, most of which encode nucleotide-binding site leucine-rich repeat (NBS-LRR, NLR) proteins (Dou and Zhou, 2012; Yuan et al., 2021a), resulting in the hypersensitive response (HR) and inhibition of pathogen infection (Alfano and Collmer, 2004; Dalio et al., 2021).

Plant hormones, including salicylic acid (SA), jasmonic acid (JA), and ethylene (ET), act as signaling molecules that mediate a diverse array of immune responses (Spoel and Dong, 2008). SA, a natural phenolic compound, regulates a wide range of immune responses triggered by PAMPs and effectors (Vlot et al., 2009; Boatwright and Pajerowska-Mukhtar, 2013). Its pivotal role in rice immunity against the blast fungus *M. oryzae* was demonstrated by generating SA-deficient transgenic rice expressing bacterial salicylate hydroxylase (*NahG*), which degrades SA (Yang et al., 2004). In *Arabidopsis thaliana*, Non-expresser of pathogenesis-related 1 (NPR1) functions as a key regulator of the SA signaling pathway, with numerous SA-responsive genes being NPR1-dependent (Dong, 2004). Overexpression of *AtNPR1* or its ortholog *OsNPR1* enhances resistance to *M. oryzae* and leaf blight pathogen *Xanthomonas oryzae* pv. *oryzae* (*Xoo*) in rice (Yuan et al., 2007; Feng et al., 2011; Dai et al., 2023). Enhanced disease susceptibility 1 (EDS1) and phytoalexin deficient 4 (PAD4) are involved in the TIR-NBS-LRR (TNL) protein-associated SA accumulation (Wiermer et al., 2005). In rice, *OsEDS1* functions as a positive regulator in rice-pathogen interactions, with knockdown of *OsEDS1* leading to increased susceptibility to *Xoo* (Ke et al., 2019). Similarly, *OsPad4* contributes to rice defense against *Xoo* (Ke et al., 2014). JA regulates plant growth, development, and biotic stress responses. JA treatment induces the expression of *pathogenesis-related* (*PR*) genes, including *OsPR10*, *OsPR5*, *OsPR1a*, and *OsPR1b* (Agrawal et al., 2000a, 2000b; Rakwal and Komatsu, 2000; Hashimoto et al., 2004), highlighting its roles in rice immunity. Overexpression of *OsAOS2*, encoding an allene oxide synthase that is a key enzyme in JA biosynthesis, leads to upregulation of *PR* gene expression and enhances resistance to rice blast.

PR proteins are well-established defense-related proteins that play critical roles in signal transduction, antimicrobial activity, and cell wall reinforcement. Various PR proteins are involved in rice immunity (Kim et al., 2009, 2013). For instance, *pathogenesis-related gene 1a* (*OsPR1a*) and *pathogenesis-related protein 10b* (*OsPR10b*) are induced upon rice blast infection (Agrawal et al., 2000a, 2000b). Phenylalanine ammonia lyases (PALs) contribute to broad-spectrum resistance against pathogens (Wang et al., 2019). In rice, there are nine *OsPAL* genes, eight of which are known to be induced by *M*. *oryzae* (Duan et al., 2014). Overexpression of *OsPAL1* enhances resistance to *M*. *oryzae* (Zhou et al. (2018), while loss of function of *OsPAL4* increases susceptibility to *M*. *oryzae*, *Xoo*, and *Rhizoctonia solani* (Tonnessen et al., 2015). Additionally, the induction of *ent-kaurene synthase 4* (*OsKS4*), *OsPBZ1*, *OsGlu1*, *OsSalT*, and *OsPR10* has been observed in rice upon *M*. *oryzae* infection (Kim et al., 2004).

In addition to *PR* genes, transcription factors, immune receptors, and ARGONAUTEs (AGOs) play essential roles in plant immunity against pathogens. Transcription factors (TFs), such as WRKY, NAC, bZIP, AP2, BHLH, NF-Y, CAMTA, and MYB, regulate the expression of downstream genes by binding to their promoters and are integral to plant immunity (Huang et al., 2016; Noman et al., 2017; Ng et al., 2018). For instance, the WRKY TF gene *OsWRKY45* plays a crucial role in SA-mediated defense signaling in rice. The expression of *OsWRKY45* is induced by SA or its analog treatment, and its overexpression enhances resistance to blast disease, whereas loss of function increases susceptibility (Shimono et al., 2007). Similarly, the NAC TF gene *NAC domain-containing protein 4* (*OsNAC4*), positively regulates HR-mediated cell death (Kaneda et al., 2009). Furthermore, immune receptor genes are critical for rice immunity. OsCERK1 and OsCEBiP, two lysin motif (LysM) proteins, are involved in the perception of chitin, a major component of the fungal cell wall that triggers innate immunity in plants (Shimizu et al., 2010; Hayafune et al., 2014). In *Arabidopsis thaliana*, chitin binding by CERK1 activates immunity, whereas in rice, CEBiP plays a dominant role in chitin perception. Knockdown of *OsCEBiP* results in the loss of chitin oligosaccharide binding to the plasma membrane, impairing OsCERK1’s ability to bind chitin directly (Kaku et al., 2006; Shinya et al., 2012). Moreover, ARGONAUTE (AGO) proteins contribute to plant responses to environmental challenges, including pathogen attacks. For example, AGO1, a key component of RNA-induced silencing complex (RISC) in the RNAi pathway, coordinates plant disease resistance with growth and development (Zhao et al., 2023).

To date, over 50 race-specific resistance genes against rice blast disease have been cloned from 17 distinct loci in rice (Huang et al., 2023). The products encoded by these genes specifically recognize corresponding avirulence effectors from *M*. *oryzae*, triggering robust immunity. Activation of these resistance genes is generally accompanied by a series of immune responses. However, it is largely unclear why different *R* genes endow differential amplitudes of immunity against *M*. *oryzae*.

In this study, we used the previously identified three elite restorer lines harboring distinct *R* genes: SH548 (carrying *Pi2*, *Ptr*, and *Pid2*), SH882 (carrying *Pid2* and *Pikm*), and WSSM (carrying *Pid2* and *Pi9*-Type5) (Hassan et al., 2022). These lines were treated with *M. oryzae* or chitin, followed by the analysis of H_2_O_2_ accumulation and the expression of defense-related genes using DAB staining and RT-qPCR, respectively. Additionally, RNA-seq data were analyzed for LTH and monogenic lines IRBLz5-CA, IRBL9-W, and IRBLkm-Ts (carrying *Pi2*, *Pi9*, and *Pikm*, respectively), as well as the blast-resistant elite restorer line R2115 (carrying *Pi2*, *Pid2*, and *Pib*), before and after inoculation with the rice blast fungus. The results indicate that different *R* genes orchestrate both convergent and divergent responses to *M. oryzae* in rice.

## Results

### Infection by *M. oryzae* resulted in differential amplitudes of immune responses in three rice restorer lines

**Figure 1:**
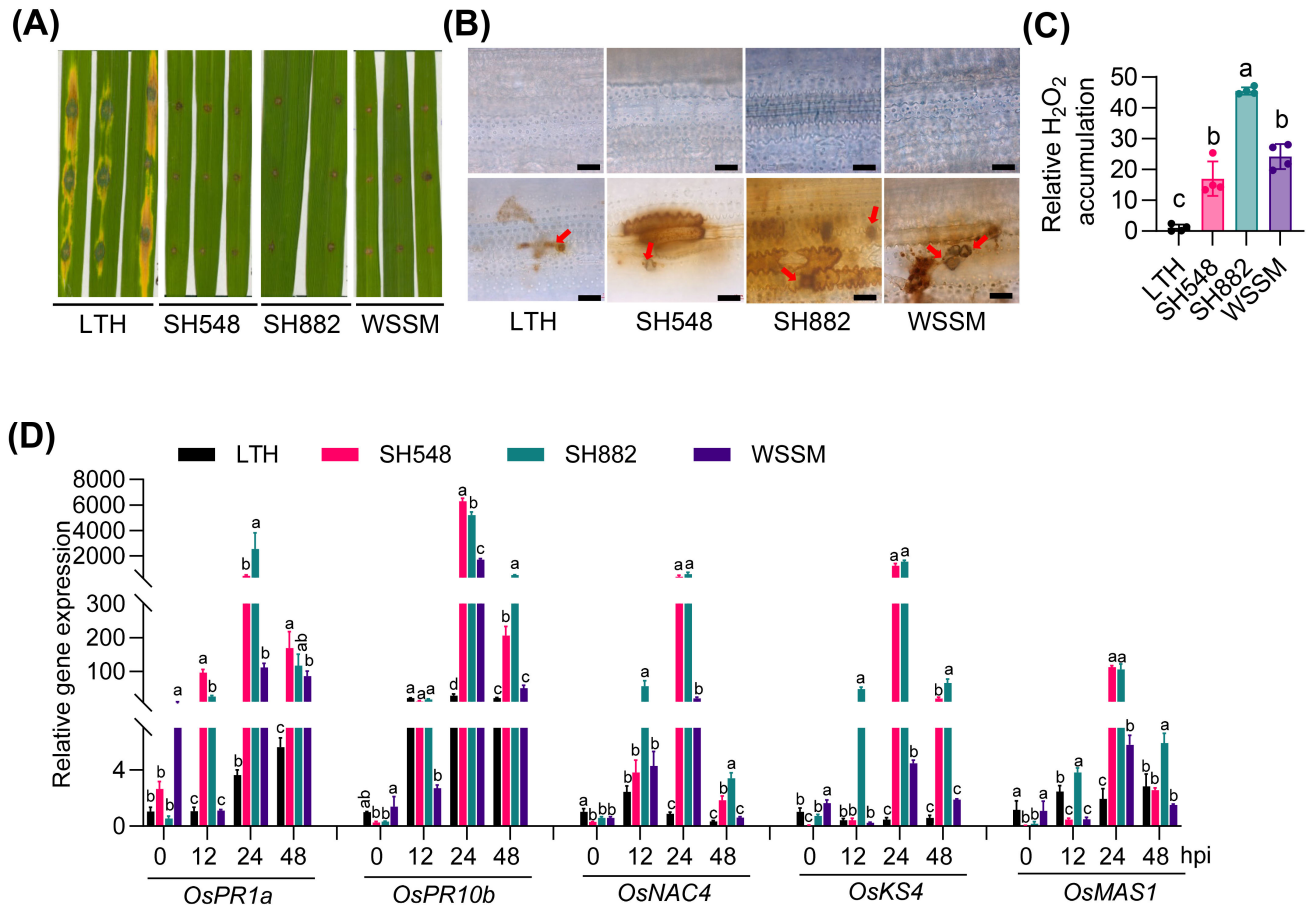
The differential amplitudes of immune responses exhibited by SH548, SH882, and WSSM upon *M. oryzae* infection. **(A)** Blast disease phenotypes in LTH, SH548, SH882, and WSSM. The indicated lines were punch inoculated with the CRB10 strain (4 × 10^5^ spores/mL), and a photograph was taken at 5 days post-inoculation (dpi). **(B)** H_2_O_2_ accumulation in LTH, SH548, SH882, and WSSM at 48 hours post-inoculation (hpi), both with and without *M. oryzae* infection. Representative leaf sections from the indicated lines were used to illustrate fungal growth and H_2_O_2_ accumulation. Brown coloration indicates H_2_O_2_ accumulation and red arrows highlight appressoria. The upper panel shows mock-treated, and the lower panel shows *M. oryzae*-infected leaves. The images were captured using a Zeiss fluorescence microscope (Zeiss imager A2). Scale bar, 10 μm. **(C)** Quantification of H_2_O_2_ in leaves of indicated rice lines infected with *M. oryzae*. **(D)** Expression patterns of defense-related genes. Leaf samples were collected at 0, 12, 24, and 48 hpi with the Guy11 (4×10^5^ spores/mL) strain. RT-qPCR was conducted to examine the expression of defense-related genes. Error bars indicate the standard deviation (SD) (n=3). Differences marked by letters indicate significant differences (P < 0.05), as determined by One-way ANOVA analysis. Differences were marked by comparing the accessions at each time point separately.

To investigate the amplitudes of immune responses in different rice accessions, we inoculated LTH and three elite restorer lines with the *M*. *oryzae* strain Guy11 and examined immune responses, including H_2_O_2_ accumulation and the expression of defense-related genes, such as *OsPR1a*, *OsPR10b*, *OsNAC4*, *OsKS4*, and *OsMAS1* (*momilactone a synthase*). Consistent with the observation in a previous report (Hassan et al., 2022), LTH was susceptible, showing typical disease lesions, while the three restorer lines exhibited resistance (**Figure 1A**). In line with the disease phenotypes, extensive H_2_O_2_ accumulation was observed as reddish-brown coloration around appressoria in the three elite restore lines, while minimal accumulation was detected in LTH after DAB staining (**Figures 1B, C**). In SH548, the expression of all examined defense-related genes was induced compared with LTH, with notably high expression levels at 24 hours post-inoculation (hpi) (**Figure 1D**). Specifically, *OsPR10b* exhibited about a 6000-fold increase in expression, *OsKS4* showed about a 1200-fold increase, and *OsPR1a* and *OsNAC4* exhibited approximately a 400-fold increase compared to LTH. *OsPR1a* and *OsNAC4* were significantly up-regulated at 12, 24, and 48 hpi, whereas *OsPR10b* and *OsKS4* exhibited increased levels at 24 and 48 hpi, and *OsMAS1* showed elevated levels specifically at 24 hpi compared to LTH. In SH882, the expression levels of *OsPR1a*, *OsNAC4*, *OsKS4*, and *OsMAS1* were significantly elevated at 12, 24, and 48 hpi, whereas *OsPR10b* exhibited enhanced expression specifically at 24 and 48 hpi compared to LTH (**Figure 1D**). Similarly, WSSM exhibited increased expression of defense-related genes at specific time points, with expression levels observed at certain time points being higher than those in LTH. Specifically, *OsMAS1* exhibited significantly higher induction than LTH at 24 hpi, whereas *OsPR1a* and *OsPR10b* displayed elevated expression at both 24 and 48 hpi, *OsNAC4* showed increased expression at 12 and 24 hpi, and *OsKS4* at 24 hpi (**Figure 1D**). These results indicate that SH548, SH882, and WSSM exhibit differential amplitudes of immune responses upon the infection of *M*. *oryzae*, despite all showing similar resistant phenotypes.

### The marker genes for different signaling pathways were differentially activated in three rice restorer lines upon *M*. *oryzae* infection

**Figure 2:**
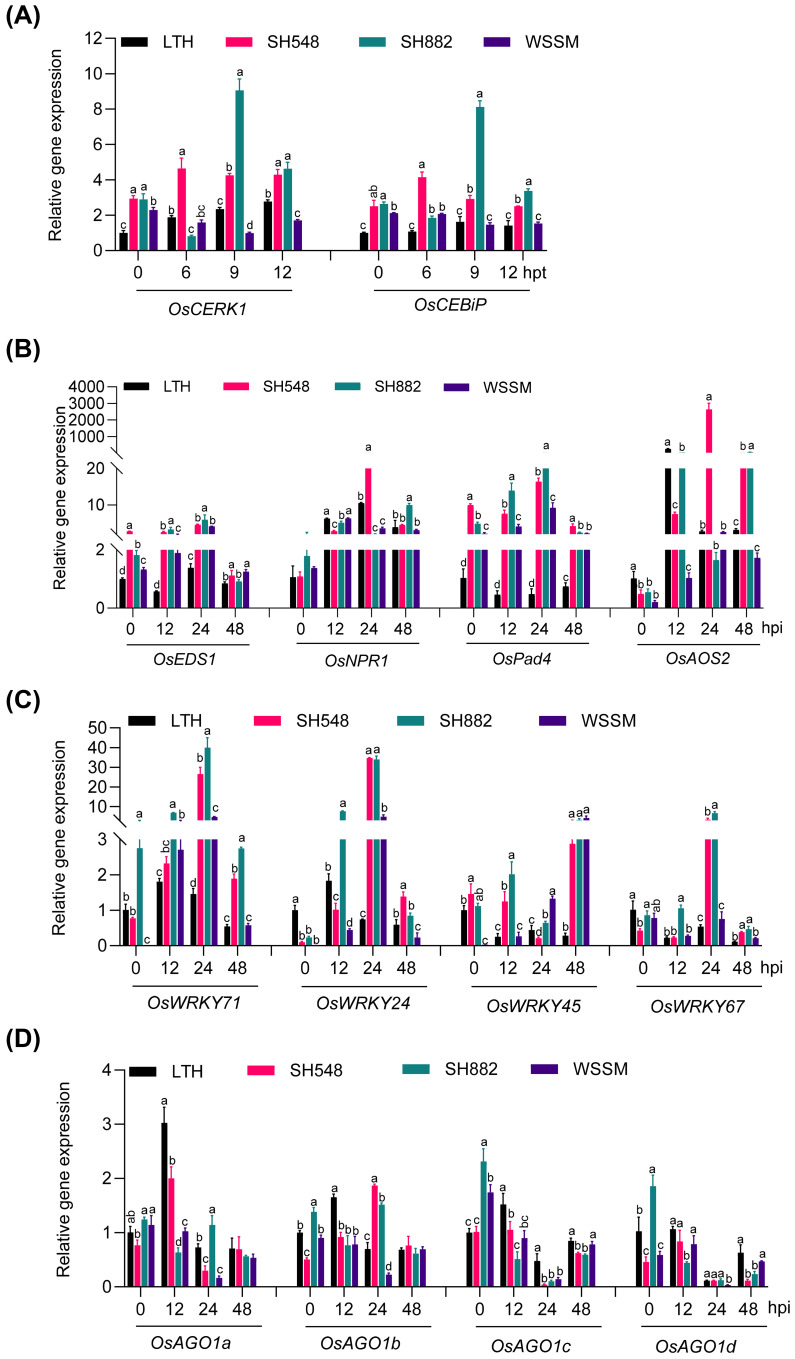
The differential response of chitin receptors upon chitin treatment and differential activation of defense-related genes upon blast infection in SH548, SH882, and WSSM. **(A)** Expression patterns of *OsCERK1* and *OsCEBiP* in LTH, SH548, SH882, and WSSM following chitin treatment. Leaves were treated with 200 μM chitin and harvested at 0, 6, 9, and 12 hours post-treatment (hpt). Error bars indicate the standard deviation (SD) (n=3). Differences marked by letters indicate significant differences (P < 0.05), as determined by One-way ANOVA analysis. Differences were marked by comparing the accessions at each time point separately. **(B-D)** Expression pattern of hormone-signaling pathway genes **(B)**, *OsWRKY* family genes **(C)**, and *OsAGO1* family genes **(D)**. Leaf samples were collected at 0, 12, 24, and 48 hpi after inoculated with the Guy11 (4×10^5^ spores/mL) strain. RT-qPCR was conducted to examine the expression of these genes. Error bars indicate the standard deviation (SD) (n=3). Differences marked by letters indicate significant differences (P < 0.05), as determined by One-way ANOVA analysis. Differences were marked by comparing the accessions at each time point separately.

To explore the reasons behind the differential amplitudes of immune responses triggered in the three resistant rice lines, we first examined whether there were any differences in the expression of the chitin receptors, including OsCERK1 and OsCEBiP, which are critical for rice immunity against *M*. *oryzae* (Hayafune et al., 2014). Previous studies have demonstrated that the expression of *CERK1* and *CEBiP* was transcriptionally induced by chitin elicitor treatment (Kaku et al., 2006; Miya et al., 2007; Shimizu et al., 2010). Our data showed that the expression of these two receptors was constitutively and significantly higher in SH548 and SH882 than in LTH, with the amplitude of *OsCEBiP* being higher in WSSM than in LTH at 6 hours post-treatment (hpt) (**Figure 2A**). Intriguingly, their expressions were differentially up-regulated in two of the three resistant lines compared to LTH upon chitin treatment. *OsCERK1* was slightly but significantly up-regulated in LTH, whereas it was highly and significantly upregulated in SH548 at 6, 9, and 12 hpt, and in SH882 at 9 and 12 hpt (**Figure 2A**). The expression of *OsCEBiP* was induced at 6 and 9 hpt in SH548, and at 9 and 12 hpt in SH882. These results indicate that different resistant lines exert diverse effects on the expression of the receptors involved in PTI upon chitin treatment.

We then examined the expression of the marker genes for the SA and JA signaling pathways upon *M*. *oryzae* infection. SA and JA are major hormones involved in plant immunity (Hou and Tsuda, 2022). SA plays a crucial role in basal resistance and is additionally involved in *R*-gene-mediated resistance, whereas JA contributes to the basal defense of rice against both fungal and bacterial pathogens (Yang et al., 2013). Transcript levels of SA signaling marker genes, including *OsEDS1*, *OsNPR1*, and *OsPad4* were induced in all three accessions at certain time points (**Figure 2B**). *OsEDS1* was induced at 12 and 24 hpi, with higher basal expression levels observed in SH548 and SH882 than in LTH (**Figure 2B**). High basal expression of *OsPad4* was observed in all the accessions, with induction occurring at 12, 24, and 48 hpi compared to LTH (**Figure 2B**). Although *OsNPR1* was significantly induced in all four accessions upon infection with *M. oryzae*, there was not a significant difference in the induction compared with LTH, except for SH548 at 24 and SH882 at 48 hpi. The JA-synthesis-related gene *OsAOS2* exhibited significant up-regulation in SH548 at 24 and 48 hpi, and in SH882 at 48 hpi, compared with LTH (**Figure 2B**). These results indicate that *M. oryzae* infection differentially activates the SA and JA signaling pathways in SH548, SH882, and WSSM.

OsWRKY proteins are known to regulate rice immunity, functioning as either positive or negative transcription factors (Jimmy and Babu, 2015). To investigate how blast infection affects the expression of *OsWRKYs* mediated by different *R* genes, we analyzed the expression patterns of *OsWRKY71*, *OsWRKY24*, *OsWRKY45*, and *OsWRKY67*, which are involved in the positive regulation of rice blast resistance (Liu et al., 2007; Shimono et al., 2007; Liu et al., 2018; Yokotani et al., 2018). *OsWRKY71* showed significant induction in SH548, SH882, and WSSM at 12 and 24 hpi (**Figure 2C**). *OsWRKY24* was induced at 24 hpi in SH548 and WSSM, and at both 12 and 24 hpi in SH882 (**Figure 2C**). *OsWRKY45*, which functions in the SA signaling pathway (Shimono et al., 2007), was up-regulated in SH548 and WSSM at 48 hpi, and in SH882 at both 12 and 48 hpi (**Figure 2C**). The expression of *OsWRKY67* was significantly up-regulated at 24 hpi in SH548 and SH882, but not up-regulated in WSSM (**Figure 2C**). These results indicate that *M*. *oryzae* infection leads to the differential up-regulation of *OsWRKY* genes, which have been previously identified as positive regulators of rice immunity.

*AGO1*, targeted by miR168 and integral to the RISC, is a core component for RNAi and is required for plant immunity (Li et al., 2019b). In rice, the *OsAGO1* gene family comprises four members: *OsAGO1a*, *OsAGO1b*, *OsAGO1c*, and *OsAGO1d*. We examined the expression patterns of these genes during rice blast infection. Compared to 0 hpi, *OsAGO1a* was induced in LTH and SH548 at 12 hpi. *OsAGO1b* was upregulated at 24 hpi in SH548 and SH882, compared with LTH. However, no up-regulation of *OsAGO1c* and *OsAGO1d* was observed in any of the three accessions (**Figure 2D**). These results indicate that *OsAGO1* is unsuitable as a marker gene for measuring the amplitude of immunity.

### Four accessions carrying different blast resistance genes exhibited both convergent and divergent transcriptome responses to blast infection

**Figure 3:**
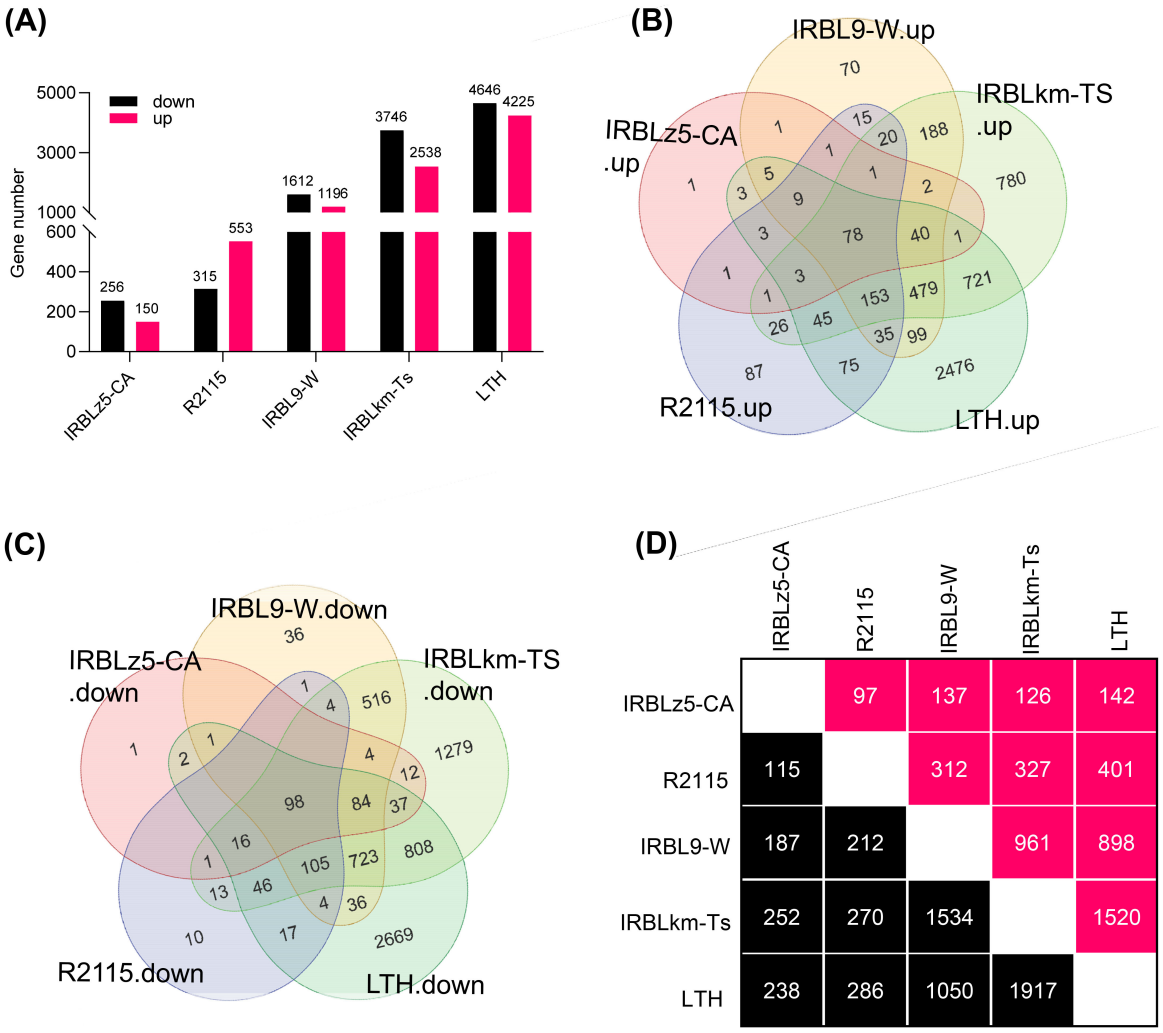
Convergent and divergent transcriptome responses exhibited by IRBLz5-CA, IRBL9-W, IRBLkm-Ts, and R2115 to blast infection. **(A)** The number of up- and down-regulated genes in all the accessions in response to blast infection. **(B, C)** Venn diagrams of up-regulated **(B)** and down-regulated **(C)** DEGs in IRBLz5-CA, IRBL9-W, IRBLkm-Ts, R2115, and LTH. Venn diagrams were generated using the R programming language. **(D)** The number of DEGs shared between each two accessions. The pink-colored boxes indicate the upregulated DEGs, whereas the black-colored boxes indicate the downregulated DEGs.

Based on the differential amplitudes of immune responses in SH548, SH882, and WSSM, we hypothesized that the different *R* genes mediate distinct transcriptional responses. To rigorously test this hypothesis, we conducted transcriptomic analysis on the blast-resistant monogenic lines IRBLz5-CA, IRBL9-W, and IRBLkm-Ts, each harboring a single *R* gene, along with the resistant elite restorer line R2115 (Shi et al., 2015) and the susceptible line LTH, both before and after infection with *M. oryzae* (Hu et al., 2023). Specifically, IRBLz5-CA harbors *Pi2*, IRBL9-W carries *Pi9*, IRBLkm-Ts contains *Pikm*, and R2115 possesses *Pi2, Pid2*, and *Pib* (Shi et al., 2015; Hu et al., 2024). Transcriptomic data analysis of infected samples detected a total of 406, 868, 2808, 6284, and 8871 differentially expressed genes (DEGs) in IRBLz5-CA, R2115, IRBL9-W, IRBLkm-Ts, and LTH (**Figure 3A**; **Supplementary Tables S1**, **S2**), respectively, with the fewest DEGs in IRBLz5-CA, followed by R2115, IRBL9-W and IRBLkm-Ts, and the most in LTH, indicating that transcriptional reprogramming response is quite different in the accessions carrying different *R* genes. Similarly, the number of up-regulated and down-regulated DEGs followed the same trend as the total DEGs, with 150, 553, 1196, 2538, and 4225 genes upregulated, and 256, 315, 1612, 3746, and 4646 genes downregulated in IRBLz5-CA, R2115, IRBL9-W, IRBLkm-Ts, and LTH, respectively (**Figure 3A**; **Supplementary Tables S1**, **S2**). Intriguingly, we detected a total of 78 upregulated and 98 downregulated DEGs shared across all five accessions, whereas 1, 87, 70, 780, and 2476 DEGs were specifically upregulated, and 1, 10, 36, 1279, and 2669 DEGs were downregulated in IRBLz5-CA, R2115, IRBL9-W, IRBLkm-Ts, and LTH, respectively (**Figures 3B, C**; **Supplementary Table S3**). Moreover, each accession shared a certain number of DEGs with other accessions (**Figure 3D**; **Supplementary Table S3**). These data indicate both convergent and divergent responses of transcriptional reprogramming upon the infection of *M*. *oryzae*.

**Figure 4:**
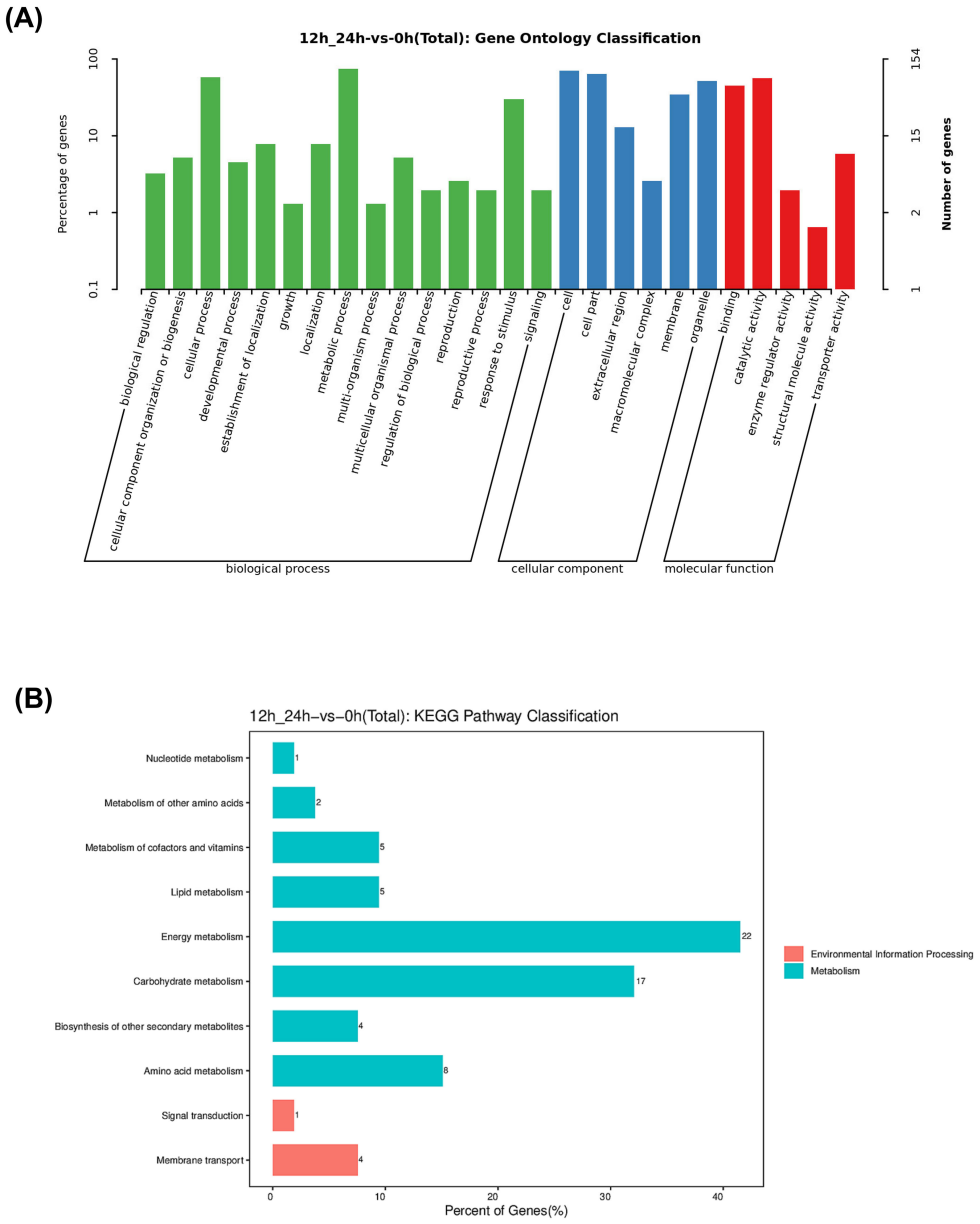
GO and KEGG pathway classification of co-up-regulated and co-down-regulated DEGs in IRBLz5-CA, IRBL9-W, IRBLkm-Ts, R2115, and LTH based on RNA-seq data. **(A)** GO classification, **(B)** KEGG pathway classification.

We then analyzed the Gene Ontology (GO) enrichment and Kyoto Encyclopedia of Genes and Genomes (KEGG) pathways of the 78 convergently upregulated and 98 downregulated DEGs. Based on GO annotations, 78 upregulated and 98 downregulated genes were categorized into three primary GO categories: biological process, cellular component, and molecular function. In the biological process category, convergent DEGs were enriched in biological regulation, cellular component organization, cellular process, and other related pathways. The most enriched categories within biological processes were cellular process, metabolic process, and response to stimulus. In the cellular component category, DEGs were enriched in the cell, cell part, extracellular region, macromolecular complex, membrane, and organelle. In the molecular function category, DEGs were enriched in binding, catalytic activity, enzyme regulator activity, structural molecule activity, and transporter activity (**Figure 4A**; **Supplementary Table S4**). KEGG pathway analysis showed that the convergent DEGs were enriched in membrane transport, signal transduction, amino acid metabolism, biosynthesis of other secondary metabolites, and other related pathways (**Figure 4B**; **Supplementary Table S4**).

Subsequently, we conducted analyses of the divergent DEGs in each rice line. In IRBL9-W, which harbors the *Pi9* resistance gene, the divergent DEGs were annotated into 28 terms, including 15 in biological processes, eight in cellular components, and five in molecular functions (**Supplementary Figure S1A**; **Supplementary Table S4**). KEGG pathway analysis revealed that the divergent DEGs in IRBL9-W were enriched in pathways such as transport and catabolism, signal transduction, folding, sorting and degradation, biosynthesis of other secondary metabolites, and other related pathways (**Supplementary Figure S1B**; **Supplementary Table S4**). In IRBLkm-Ts, which carries the *Pikm* resistance gene, the divergent DEGs were annotated into 28 terms, including 15 in biological processes, eight in cellular components, and five in molecular functions (**Supplementary Figure S2A**; **Supplementary Table S4**). KEGG pathway analysis revealed that these divergent DEGs were enriched in pathways related to cell motility, transport and catabolism, membrane transport, signal transduction, and other related pathways (**Supplementary Figure S2B**; **Supplementary Table S4**). In R2115, the divergent DEGs were annotated into 26 terms, including 14 in biological processes, eight in cellular components, and four in molecular functions (**Supplementary Figure S3A**; **Supplementary Table S4**). KEGG pathway analysis showed enrichment of the divergent DEGs in signal transduction, biosynthesis of other secondary metabolites, carbohydrate metabolism, lipid metabolism, and other pathways (**Supplementary Figure S3B**; **Supplementary Table S4**). In LTH, which does not harbor any blast resistance genes, the divergent DEGs were annotated into 28 terms, including 15 in biological processes, eight in cellular components, and five in molecular functions (**Supplementary Figure S4A**; **Supplementary Table S4**). KEGG pathway analysis revealed enrichment of the divergent DEGs in pathways related to cell motility, transport and catabolism, membrane transport, signal transduction, and other pathways (**Supplementary Figure S4B**; **Supplementary Table S4**). Notably, the distribution of genes across each term varies significantly among the accessions harboring different *R* genes.

Altogether, these data indicate that rice plants mount convergent responses to the infection of *M*. *oryzae* regardless of blast resistance genes, whereas different *R* genes endow the divergent responses.

### Transcriptome profiling confirmed the divergent and convergent DEGs associated with crucial immune pathways in the accessions carrying different *R* genes

**Figure 5:**
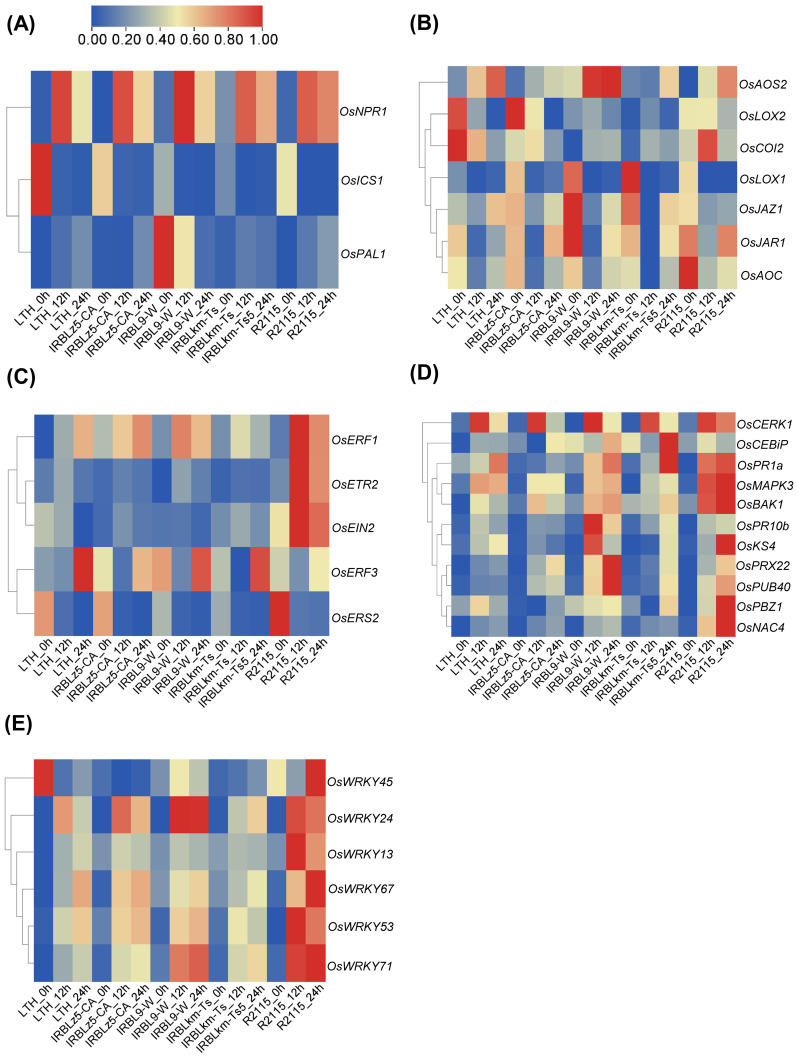
Heat maps displaying DEGs related to plant immunity in IRBLz5-CA, IRBL9-W, IRBLkm-Ts, R2115, and LTH upon blast infection. The differential expression of genes involved in the SA-signaling pathway **(A)**, JA-signaling pathway **(B)**, ET-signaling pathway **(C)**, PTI pathway **(D)**, and *OsWRKY* family **(E)** genes was analyzed upon *M. oryzae* infection. Each row of transcriptome data was normalized using TBtools.

To investigate the reasons behind the differential amplitudes of immune responses triggered by different *R* genes, we focused on the divergent and convergent DEGs associated with crucial immune pathways. Our analysis unveiled diverse expression patterns of defense-related and hormone-signaling pathway genes in IRBLz5-CA, IRBL9-W, IRBLkm-Ts, R2115, and LTH (**Figure 5**). In the SA signaling pathway, *OsNPR1* exhibited high induction levels across all accessions. However, *OsICS1* and *OsPAL1* showed only marginal changes in all accessions, except for the high basal expression of *OsICS1* in IRBLz5-CA, R2115, and LTH, and of *OsPAL1* in IRBL9-W (**Figure 5A**). In the JA pathway, *OsAOS2* exhibited high induction in IRBL9-W and R2115 at 12 and 24 hpi, with a slight induction observed at 24 hpi in IRBLz5-CA and IRBLkm-Ts. Compared with other accessions, the transcript level of *OsCOI2* was significantly induced in R2115 at 12 hpi. High basal expression levels of *OsLOX1*, *OsJAZ1*, *OsJAR1*, and *OsAOC* were observed in IRBLz5-CA, IRBL9-W, IRBLkm-Ts, and R2115, while *OsLOX2* exhibited high basal expression in IRBLz5-CA, R2115, and LTH (**Figure 5B**). In the ET signaling pathway, *OsERF1* exhibited significant induction in all the accessions, except IRBLkm-Ts, which showed a slight induction at 12 hpi. High expression levels of *OsETR2* and *OsEIN2* were observed only in R2115 at 12 and 24 hpi. *OsERF3* was significantly induced in all the accessions at 24 hpi, except R2115 (**Figure 5C**). High basal expression levels of *OsERS2* were observed in all the accessions. These results indicate the differential upregulation of hormone signaling in all four accessions upon *M. oryzae* infection.

Next, in the PTI signaling genes, the pattern recognition receptor *OsCEBiP* was induced at 24 hpi in IRBLz5-CA, IRBL9-W, and IRBLkm-Ts and at 12 hpi in R2115, whereas *OsCERK1* was upregulated in IRBLz5-CA, IRBL9-W, and IRBLkm-Ts at 12 hpi, and in R2115 at both 12 and 24 hpi (**Figure 5D**). Compared with the susceptible control LTH, enhanced induction of *OsBAK1* was observed in IRBLkm-Ts at 24 hpi, and in IRBLz5-CA, IRBL9-W, and R2115 at both 12 and 24 hpi. *OsMAPK3* exhibited slight induction in IRBLz5-CA, IRBL9-W, and IRBLkm-Ts at 12 and 24 hpi, but showed enhanced induction in R2115 at 12 and 24 hpi. *OsKS4* was induced at 12 and 24 hpi in IRBL9-W and R2115, respectively. *OsPR10b* exhibited higher induction levels in IRBL9-W at 12 hpi, compared to other accessions. *OsPR1a* induction was enhanced in IRBLkm-Ts at 24 hpi and at 12 and 24 hpi in IRBL9-W and R2115. *Probenazole-induced protein 1* (*PBZ1*), a well-established marker gene for cell death in rice (Kim et al., 2008), showed a slight induction in IRBLkm-Ts at 24 hpi, followed by a remarkable induction in R2115 at 24 hpi. Both *OsPRX22* and *OsPUB40* were induced in IRBL9-W and R2115 at 12 and 24 hpi. Transcript levels of *OsNAC4* were notably induced in R2115 at 12 and 24 hpi, with slight induction detected in other accessions. Significant induction of *OsWRKY24*, *OsWRKY53*, *OsWRKY67*, and *OsWRKY71* was observed in all four resistant accessions. *OsWRKY13* and *OsWRKY45* were specifically induced in R2115 (**Figure 5E**). Again, these data indicate the convergent and divergent responses across all accessions, each carrying different *R* genes.

**Figure 6:**
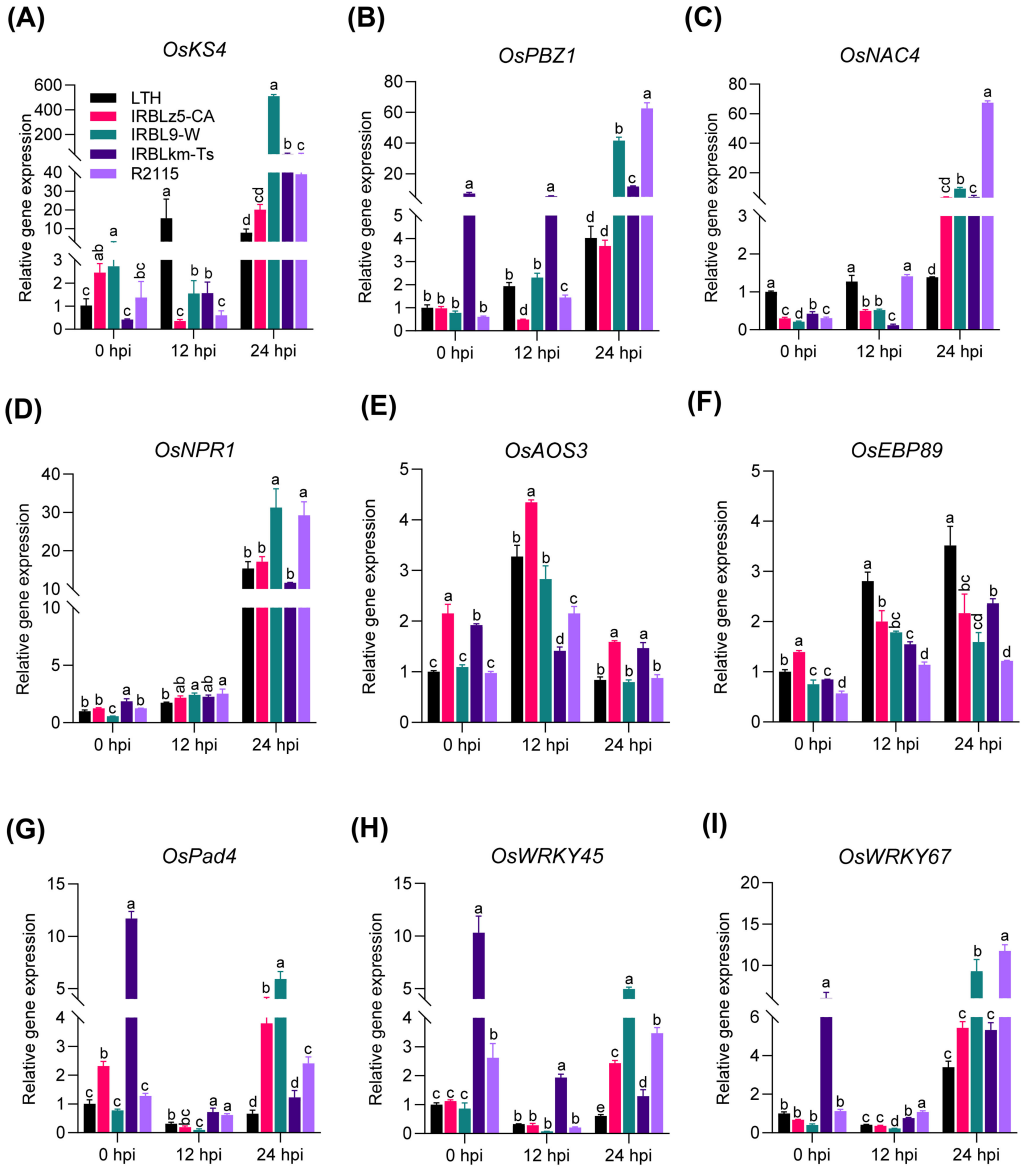
The expression patterns of defense-related genes and hormone-signaling pathway genes in LTH, IRBLZ5-CA, IRBL9-W, IRBLKM-Ts, and R2115. All four accessions showed induced expression of defense-related genes **(A–C, F, H, I)** and hormone-signaling pathway genes **(D, E, G)** compared with LTH. Leaf samples were collected at 0, 12, and 24 hpi with the Guy11 (4×10^5^ spores/mL). Error bars indicate the standard deviation (SD) (n=3). Differences marked by letters indicate significant differences (P < 0.05), as determined by One-way ANOVA analysis. Differences were marked by comparing the accessions at each time point separately.

To further validate the conclusions drawn from the RNA-seq result, we validated the expression of convergently upregulated DEGs involved in defense and hormone signaling pathways by RT-qPCR. The expression of *OsKS4*, *OsPBZ1*, and *OsNAC4* was significantly induced in IRBL9-W, IRBLkm-Ts, and R2115 at 24 hpi, compared with LTH (**Figures 6A-C**). The expression of *OsNPR1* and *OsWRKY67* was significantly induced in IRBL9-W and R2115 at 24 hpi, compared with LTH (**Figures 6D, I**). *OsAOS3* was significantly induced to higher levels at 12 hpi than at 0 hpi in IRBLz5-CA, IRBL9-W, and R2115 (**Figure 6E**), whereas *OsEBP89* was induced at 12 and 24 hpi in all the accessions. Although the magnitude of induction was not as high as that of LTH, the expression was still upregulated compared to 0 hpi (**Figure 6F**). A significant induction of *OsPad4* was observed in IRBLz5-CA, IRBL9-W, and R2115 at 24 hpi and *OsWRKY45* in all the accessions at 24 hpi, compared with LTH, accompanied by a high basal expression level in IRBLkm-Ts (**Figures 6G, H**). Thus, these results indicate that the RNA-seq data are reliable, and various blast resistance genes mount differential amplitudes of immune responses via differentially up-regulating genes involved in multiple immune pathways.

**Figure 7:**
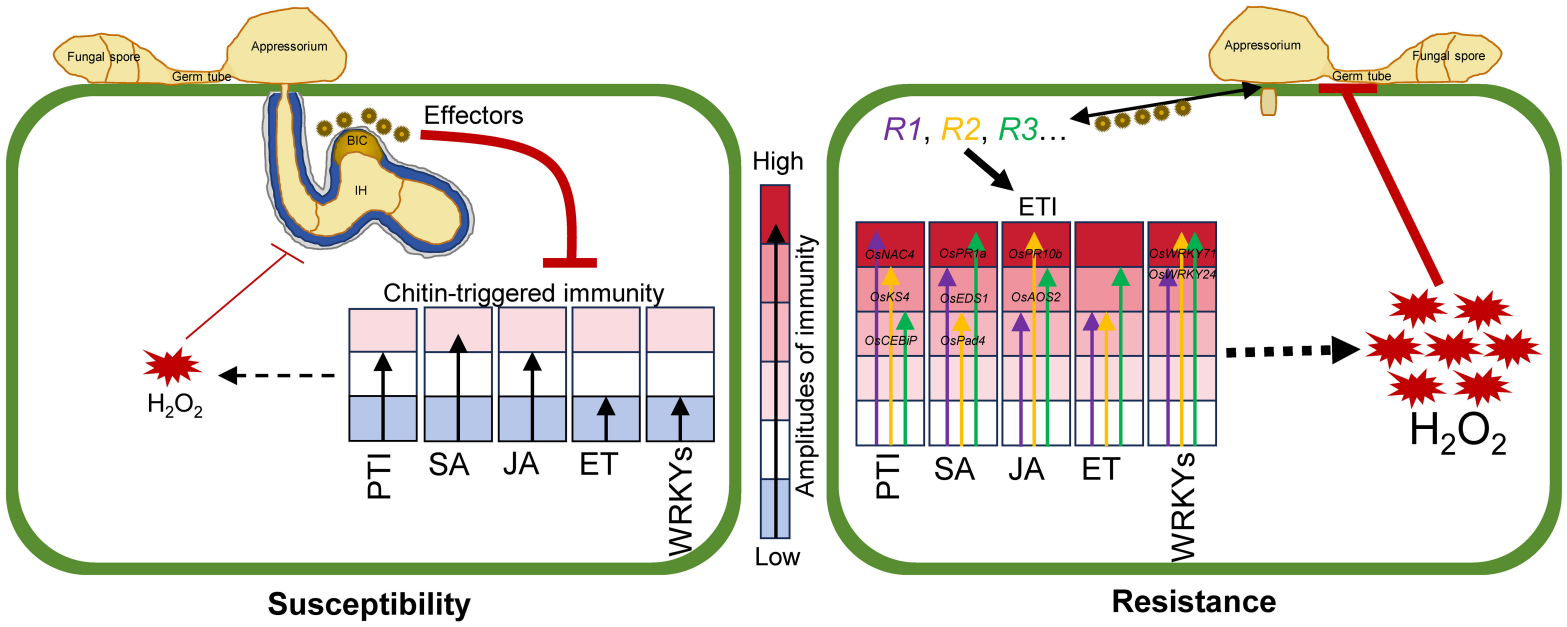
Working model for different *R* genes endow with differential amplitudes of defense against *M*. *oryzae.* Secretion of *M. oryzae* effectors to rice cells leads to the suppression of chitin-triggered immunity, which results in weak H_2_O_2_ accumulation and a low amplitude of defense against *M. oryzae*, thereby triggering susceptibility. However, the recognition of effectors by different *R* genes leads to the activation of downstream signaling pathways with differential amplitudes, including increased H_2_O_2_, and activation of PTI, SA, JA, ET, and WRKY pathways, resulting in enhanced resistance. *R1*, *R2*, *R3*: Resistance genes. Different-sized arrows: The differential amplitude of immunity endowed by each *R* gene. BIC: Biotrophic interfacial complex. IH: Invasive hyphae.

We propose a working model in which different *R* genes confer differential amplitudes of defense against *M*. *oryzae* (**Figure 7**). In the absence of *R* genes, weak H_2_O_2_ accumulation and a low amplitude of defense against *M. oryzae* occur, as more effector proteins are secreted into the cells. In contrast, the recognition of effectors by different *R* genes leads to increased H_2_O_2_ accumulation and activation of PTI, SA, JA, and WRKY pathways (**Figure 7**).

## Discussion

### Different blast *R* genes mount differential amplitude of immunity against *M*. *oryzae*

Our results revealed divergent immune responses exhibited by the susceptible accession LTH and resistant accessions SH548, SH882, WSSM, IRBLZ5-CA, IRBL9-W, IRBLKM-TS, and R2115 in response to blast infection. Moreover, accessions carrying broad-spectrum blast resistance genes show the activation of immune signaling pathways, though the extent to which the *R* genes regulate immunity varies among accessions. This indicates that the different *R* genes regulate the immune responses differently. However, the activation of downstream defense- and hormone-signaling pathways mediated by rice NLRs remains to be further investigated. In this study, we demonstrated that *M. oryzae* triggered differential amplitudes of H_2_O_2_ accumulation and blast infection-induced marker genes associated with different defense and hormone signaling pathways in LTH and the resistant accessions carrying different *R* genes (**Figures 1**, **2**, **5**, **6**). Therefore, this study provides valuable insights into the role of *R* genes in activating downstream defense and hormone signaling pathways in response to blast infection.

PTI and ETI share several convergent downstream responses, including calcium flux, reactive oxygen species (ROS), MAPK cascades, callose deposition, and production of phytohormones such as JA, ET, and SA (Tsuda and Katagiri, 2010; Cui et al., 2015; Peng et al., 2018). Upon pathogen attack, a complex array of signaling pathways is activated, modulating pathogen-induced resistance through a sophisticated signal transduction network (Ding et al., 2022). LTH is susceptible to over 1,000 isolates worldwide, and no functional *R* genes have been identified (Yang et al., 2022a), indicating the inactivation of ETI in LTH. This partially explains the differential defense response amplitudes between LTH and the other accessions carrying *R* genes.

### The amplitude of immunity can be measured by H_2_O_2_ and the expression of a group of marker genes

Pathogen-induced H_2_O_2_ accumulation plays a crucial role in rice disease resistance (Li et al., 2019a). Notably, our results showed increased H_2_O_2_ accumulation in SH548, SH882, and WSSM, each harboring broad-spectrum blast *R* genes (Hassan et al., 2022). Although the three elite restorer lines showed similar resistant phenotypes, the H_2_O_2_ amounts were more than that in LTH and varied among them (**Figure 1**). The highest H_2_O_2_ accumulation was observed in SH882, followed by WSSM and SH548 (**Figures 1B, C**), implying that the NLR-encoding *R* genes differentially contribute to the spatiotemporal H_2_O_2_ accumulation, thereby limiting *M. oryzae* growth at the infection site. However, it is unknown how this *R* gene-mediated downstream signaling contributes to H_2_O_2_ accumulation. A previous study has demonstrated that Osa‐miR398b enhances H_2_O_2_ production and rice blast disease resistance by modulating multiple superoxide dismutases (Li et al., 2019a). Proteomic studies on the rice and *M. oryzae* interaction identified key ROS-related proteins involved in pathogen recognition and contributing to rice resistance (Meng et al., 2019). Various ROS-scavenging enzymes, such as OsPRX59 and OsPRX62, accumulate in incompatible interactions between rice and *M*. *oryzae* (Lin et al., 2018). Cao et al. (2016) identified increased accumulation of the rice NADPH oxidase OsRBOH8 in a PM proteomics study using rice leaves collected 48 hours post-inoculation with *M. oryzae*. Whether the *R* genes in the three restorer accessions control H_2_O_2_ accumulation through the aforementioned signaling pathways remains unknown and requires further investigation.

The activation of downstream signaling via NLRs is a complex process, with varying degrees of activation. In the context of multiple sensor NLR-mediated immune responses, *NRG1* and *ADR1* act as helper NLRs (Saile et al., 2020). They are involved not only in the induction of cell death in various other NLRs (Dong et al., 2016; Castel et al., 2019) but also cooperate with EDS1, senescence-associated gene 101 (SAG101), and PAD4 to activate TNL-mediated immunity (Sun et al., 2021). The transcription of *OsEDS1* and *OsPad4* was strongly induced by blast fungus and differed significantly among the three restorer accessions (**Figure 2B**), indicating that the two genes could be used as the marker genes to measure SA-related immune responses (**Figure 7**).

The perception of chitin by OsCERK1 and OsCEBiP triggers the activation of various immune responses critical for rice immunity against *M*. *oryzae* (Hayafune et al., 2014). Consistent with the opinion that PTI and ETI mutually enhance each other to trigger robust disease resistance (Ngou et al., 2021; Yuan et al., 2021b), the defense-related genes were up-regulated to higher levels in the three restorer lines than that in LTH (**Figure 1D**), in which pathogen-induced ETI is absent because of the lack of functional *R* genes. Consistently, the expression of *OsCERK1* and *OsCEBiP* was constitutively higher in the three restorer accessions than that in LTH, and induced to higher levels by chitin in SH548 and SH882 (**Figure 2A**), and by *M. oryzae* in IRBLZ5-CA, IRBL9-W, IRBLKM-TS, and R2115 (**Figure 5D**). As the *OsCEBiP* was significantly induced in SH548 and SH882 compared with LTH, with a weak induction in LTH compared to *OsCERK1*, it could be used as a marker to measure the amplitudes of PTI responses in rice (**Figure 7**). Besides, as *OsNAC4* and *OsKS4* are involved in PTI, and these two genes were significantly upregulated to higher levels in the three restorer lines than in LTH, therefore these genes could be used as marker genes to measure the amplitudes of PTI responses in rice (**Figure 7**).

Hormone-related marker genes were also induced by *M. oryzae* in the three restorer accessions. For example, *PR10b* was induced by *M*. *oryzae* through the activation of JA signaling (Hashimoto et al., 2004). In all the accessions, induced expression of *OsPR10b* and *OsAOS2* was observed, indicating the activation of JA signaling (**Figures 1D**, **2B**, **5B, D**) by *M. oryzae*. Moreover, both *OsPR10b* and *OsAOS2* were induced to significantly higher levels at 24 or 48 hpi of *M. oryzae* in the three restorer accessions than in LTH, indicating that the two genes could be used as JA signaling pathway-related defense amplitude marker genes (**Figure 7**).

In rice, the SA pathway is regulated by *OsNPR1* (De Vleesschauwer et al., 2014). Ectopic expression of *NPR1* in rice was associated with constitutive *PR* transcripts’ expression, resulting in enhanced resistance to blast (Chern et al., 2005; Yuan et al., 2007; Sugano et al., 2010). A previous report showed that 12 rice *OsPR1* genes were upregulated upon blast infection (Mitsuhara et al., 2008). Consistently, *OsPR1a* was remarkably induced to higher levels by *M. oryzae* in the *R* gene-carried accessions, except for IRBLz5-CA, compared to LTH. However, *OsNPR1* was also induced by *M. oryzae* in LTH, with similar or higher mRNA amounts compared to those in the restorer accessions (**Figures 2B**, **6D**). These results indicate that *OsPR1a*, rather than *OsNPR1*, could serve as a marker gene to indicate defense amplitudes (**Figure 7**).

WRKY transcription factors are widely involved in regulating development, growth, and defense responses to abiotic or biotic stresses in rice (Wei et al., 2013; Jeyasri et al., 2021). Consistent with a previous finding (Yokotani et al., 2018), *OsWRKY24* was induced by *M. oryzae* in all the resistant accessions, whereas *OsWRKY71* was upregulated in all accessions as well, accompanied by the highest induction in SH882 at all time points and the lowest induction levels in LTH (**Figures 2C** and **5E**). Therefore, *OsWRKY71* and *OsWRK24* could be used as defense amplitude marker genes (**Figure 7**).

Altogether, we recommended 10 genes for measuring the amplitude of immunity against *M*. *oryzae* mediated by different blast *R* genes (**Figure 7**), providing convenience for examining the intensity of immune responses in rice.

### *R* gene-mediated differential amplitude of immunity associates with the divergent response of transcriptional reprogramming

Susceptible cultivars typically exhibit a greater number of DEGs following pathogen infection compared to resistant cultivars. A comprehensive transcriptome analysis was conducted on resistant and susceptible rice accessions following blast infection, uncovering a substantial number of convergent and divergent DEGs, along with genes associated with rice stress responses (**Figure 3**). Our RNA-seq data analysis revealed that the greatest number of DEGs was observed in LTH after blast infection, compared with the resistant accessions (**Figure 3A**). This finding aligns with previous studies. For example, Kumar et al. (2021) reported a greater number of DEGs in the blast-susceptible cultivar HP2216 than in the blast-resistant cultivar Tetep. Similarly, Yang et al. (2022b) observed a greater number of DEGs in the rice sheath blight-susceptible cultivar Koshihikari compared with the resistant cultivar Shennong 9819. Furthermore, Zhang et al. (2017) also observed a greater number of DEGs in the susceptible cultivar Lemont compared with the moderately resistant cultivar TeQing in response to rice sheath blight. These findings, in conjunction with our results, suggest that pathogen infection significantly alters the global gene expression profiles of plants, with a more pronounced effect in susceptible plants. This heightened impact in susceptible plants may be attributed to their increased energy expenditure on stress management, resulting in reduced growth and yield. Moreover, the higher number of DEGs in susceptible plants may also be due to the greater infection pressure they experience. Therefore, it seems that the number of DEGs is reversely correlated with the resistance and defense amplitudes.

## Materials and methods

### Plant growth conditions

Rice accessions used in this study included IRBLz5-CA, IRBL9-W, IRBLkm-Ts, Yahui2115 (R2115), Lijiangxin Tuan Heigu (LTH), Shu Hui 548 (SH548), Shu Hui 882 (SH882), and Wu Shan Si Miao (WSSM). All accessions were grown in a growth chamber under a photoperiod cycle of 12 hours of light and 12 hours of dark, at 26°C and 70% relative humidity.

### Pathogen inoculation and chitin treatment

For rice blast inoculation, *M. oryzae* strains Guy11 (carrying *Avr*-*Pita*) (Xiao et al., 2024) and CRB10 (carrying *Avr*-*Piz*, *Avr*-*Pita2*, and *Avr*-*Pik^s^*) (Fang et al., 2018) were cultured on the oatmeal tomato agar (OTA) medium for two weeks under a photoperiod cycle of 12 hours of light and 12 hours of dark. Subsequently, the hyphae were scratched to promote sporulation. The CRB10 strain (4 × 10^5^ spores/mL) was used for punch inoculation assay on SH548, SH882, WSSM, and LTH, whereas the Guy11 strain (4 × 10^5^ spores/mL) was used for spray inoculation on all accessions, including LTH, IRBLz5-CA, IRBL9-W, IRBLkm-Ts, R2115, SH548, SH882, and WSSM at the three-leaf stage. Besides, the leaves of LTH, SH548, SH882, and WSSM were inoculated with 200 μM chitin in 0.1 mmol/L 6-benzyladenine buffer, and samples were collected at 0, 6, 9, and 12 hpt. All the samples underwent subsequent RNA extraction and RT-qPCR analysis.

### 3,3’-diaminobenzidine staining and microscopy analysis

DAB staining was performed by following an established procedure (Zhao et al., 2020). Briefly, three-week-old seedlings of LTH, SH548, SH882, and WSSM were inoculated with Guy11 (4 × 10^5^ spores/mL). Leaves of each accession were collected at 48 hpi and immersed in 10 mL tubes filled with DAB solution, prepared by dissolving DAB in HCl and adjusting the pH to 3.8. The leaf samples were then vacuum-infiltrated for 30 minutes and incubated in darkness overnight. Subsequently, the leaves were washed with 95% ethanol and continuously washed in a 65^°^C water bath until they turned colorless. H_2_O_2_ accumulation and fungal structures within the leaves were examined using a fluorescence microscope (Zeiss imager A2).

### RNA-seq data analysis

The RNA-seq data used in this study were derived from a previous investigation (Hu et al., 2023).

For each accession, we defined upregulated genes as those that were upregulated at 12 hours but not downregulated at 24 hours, and those upregulated at 24 hours but not downregulated at 12 hours. Similarly, we identified downregulated genes using the same criteria in reverse. Subsequently, R programming language was employed to conduct overlap analyses of the upregulated and downregulated gene sets in each accession and to generate Venn diagrams, along with GO and KEGG pathway classifications. For the analysis of the relative expression of plant immune-related genes, Log2RPKM (reads per kilobase per million) values from two biological replicates of transcriptome data were used. Normalization of each row of transcriptome data and heat map generation were performed using TBtools.

### RNA extraction and RT-qPCR analysis of gene expression

Total RNA was extracted from the leaves using VeZol reagent (Vazyme), and its concentration was measured using a Nanodrop 2000 spectrophotometer (Thermo Scientific). Subsequently, cDNA was synthesized using NovoScript Plus All-in-one 1^st^ Strand cDNA Synthesis SuperMix (Novoprotein, China). RT-qPCR was conducted with AceQ Universal SYBR qPCR Master Mix (Vazyme, China) to analyze the expression patterns of defense-related genes, hormone signaling pathway genes, and *OsAGO1* family genes. Gene expression levels were normalized using *Ubiquitin* (*Ubi*) as an internal control. Primers used in this study are listed in **Supplementary Table S5**.

## Data Availability

The datasets presented in this study can be found in online repositories. The names of the repository/repositories and accession number(s) can be found in the article/**Supplementary Material**.

## Supplementary material

Supplementary Figure 1 GO and KEGG pathway classification of up-regulated and down-regulated DEGs in IRBL9-W. **(A)** GO classification, **(B)** KEGG pathway classification.

Supplementary Figure 2 GO and KEGG pathway classification of up-regulated and down-regulated DEGs in IRBLkm-Ts. **(A)** GO classification, **(B)** KEGG pathway classification.

Supplementary Figure 3 GO and KEGG pathway classification of up-regulated and down-regulated DEGs in R2115. **(A)** GO classification, **(B)** KEGG pathway classification.

Supplementary Figure 4 GO and KEGG pathway classification of up-regulated and down-regulated DEGs in LTH. **(A)** GO classification, **(B)** KEGG pathway classification.
