# Supplementary material for: Divergent response associates with the differential amplitudes of immunity against Magnaporthe oryzae by different blast resistance genes
Source: Front Plant Sci. 2025 Feb 24;16:1547593. doi: 10.3389/fpls.2025.1547593 (PMC11891227; doi:10.3389/fpls.2025.1547593)
Supplement: Supplementary Figure 1 — GO and KEGG pathway classification of up-regulated and down-regulated DEGs in IRBL9-W. (A) GO classification, (B) KEGG pathway classification. [file DataSheet1.zip › Data Sheet 1aaa/Supplementary files/Supplementary figures-2024.12.12.pptx]

## Slide 1
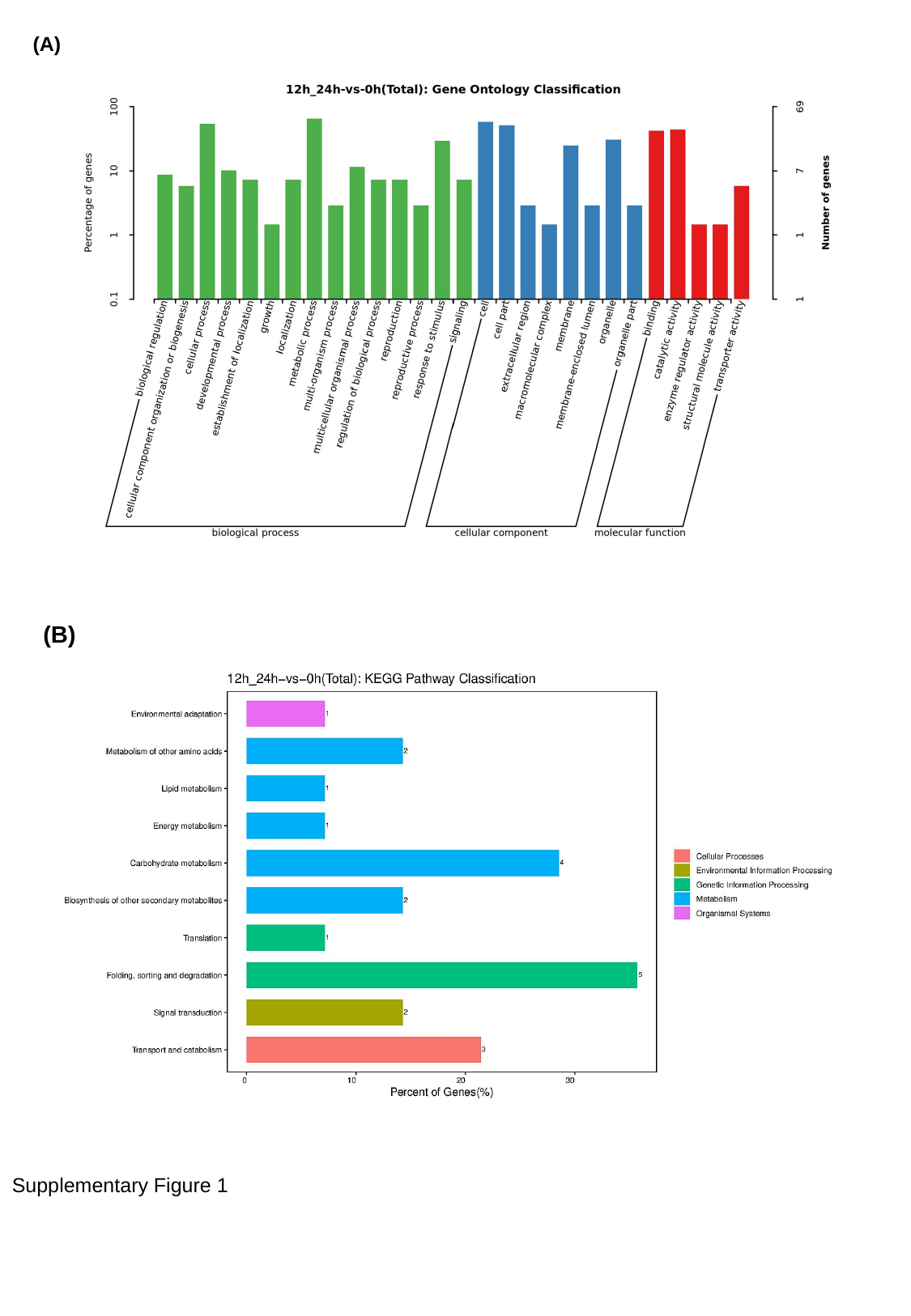

(A)
(B)
Supplementary Figure 1

## Slide 2
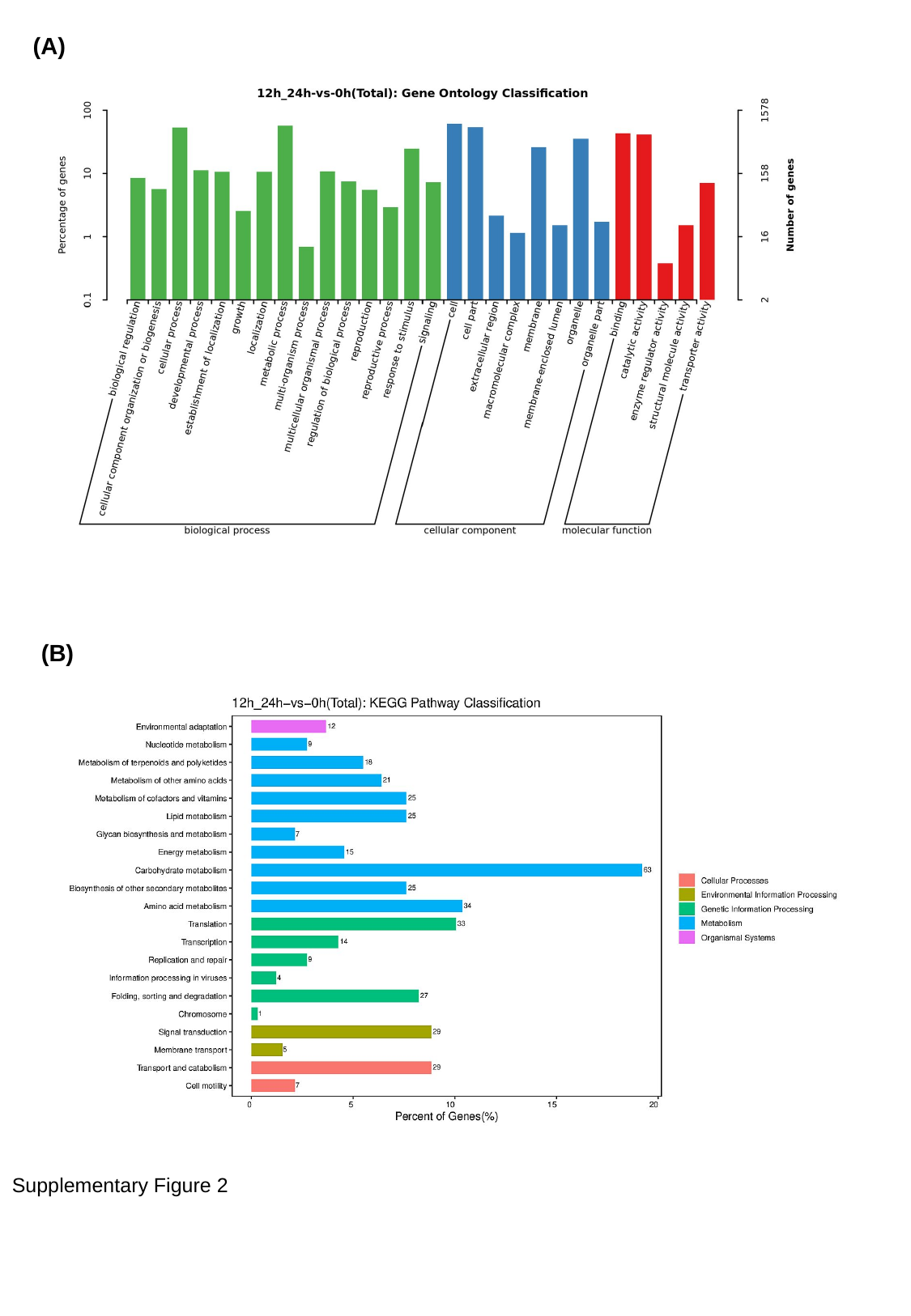

(A)
(B)
Supplementary Figure 2

## Slide 3
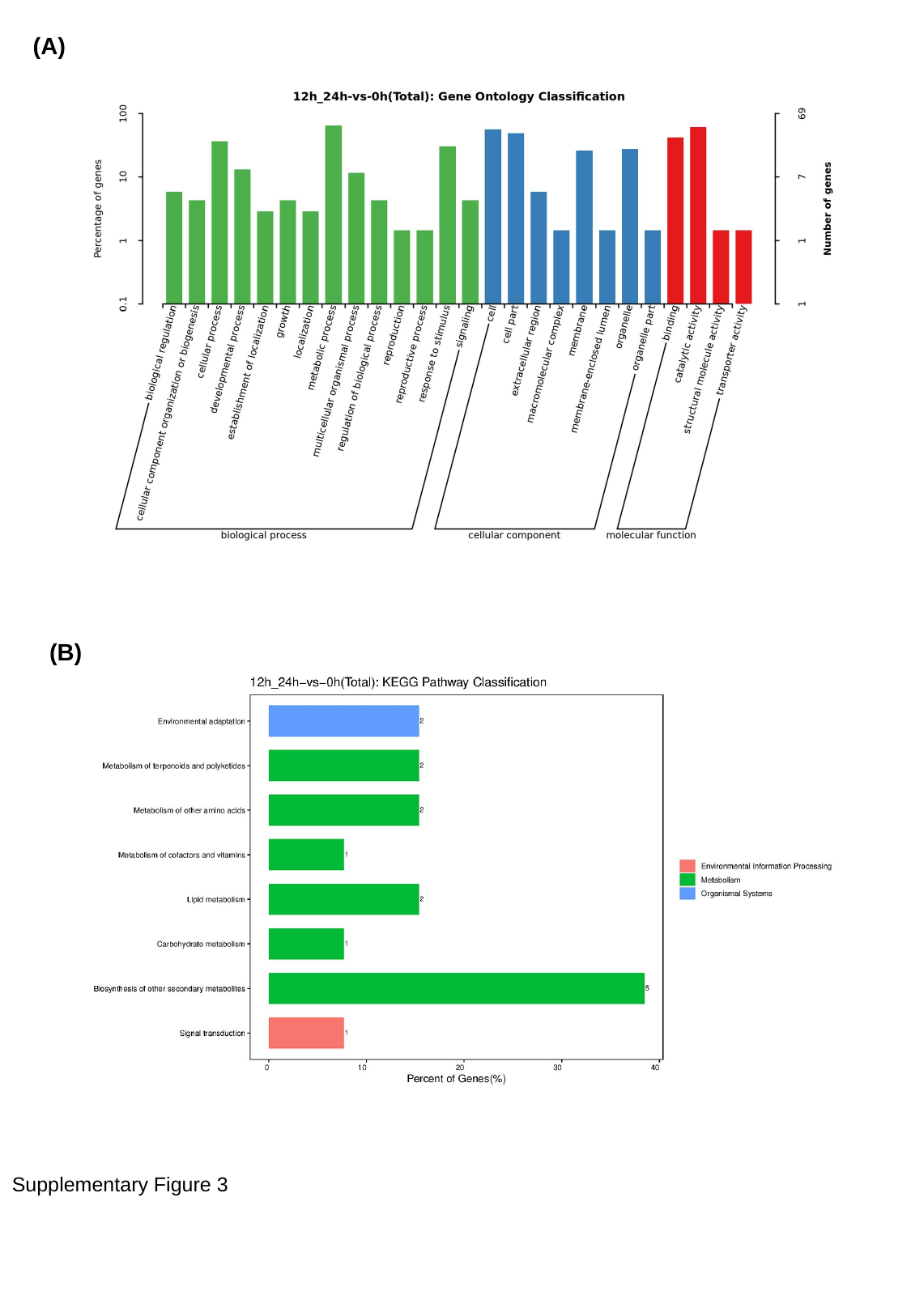

(A)
(B)
Supplementary Figure 3

## Slide 4
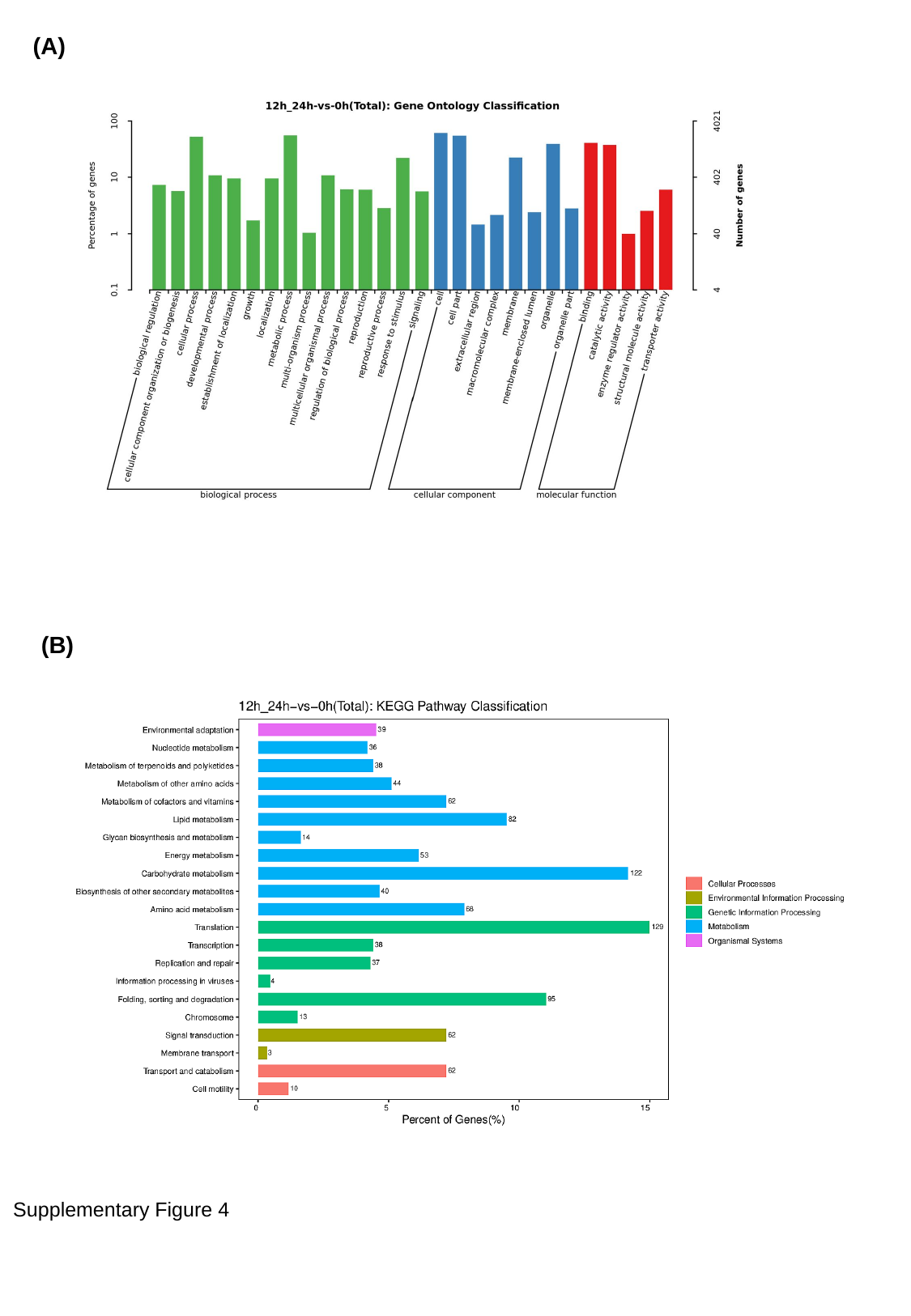

(A)
(B)
Supplementary Figure 4
