# Supplementary material for: Divergent response associates with the differential amplitudes of immunity against Magnaporthe oryzae by different blast resistance genes
Source: Front Plant Sci. 2025 Feb 24;16:1547593. doi: 10.3389/fpls.2025.1547593 (PMC11891227; doi:10.3389/fpls.2025.1547593)
Supplement: Supplementary Figure 1 — GO and KEGG pathway classification of up-regulated and down-regulated DEGs in IRBL9-W. (A) GO classification, (B) KEGG pathway classification. [file DataSheet1.zip › Data Sheet 1aaa/Supplementary files/Supplementary Table S5.docx]

**Supplementary Table S5** Primers used in this study.

| **Primer name** | **Sequence (5’-3’)** | **Annotation** |
| --- | --- | --- |
| Ubi-F | GCCCAAGAAGAAGATCAAGAAC | RT-qPCR |
| Ubi-R | AGATAACAACGGAAGCATAAAAGTC | RT-qPCR |
| OsEDS1-F | CATTCCAAGAACGAGGACACTG | RT-qPCR |
| OsEDS1-R | CAAGACTCAAGGCTAGAACCGA | RT-qPCR |
| OsPad4-F | CGGTGCTTTGTGTCACATTC | RT-qPCR |
| OsPad4-R | GACATTGAGTGGGCAGAAGA | RT-qPCR |
| OsNPR1-F | CGTCTCCTTGATGTCCTTGATAA | RT-qPCR |
| OsNPR1-R | GGTTTGACCGGACTACCATATC | RT-qPCR |
| OsAOS2-F | CAATACGTGTACTGGTCGAATGG | RT-qPCR |
| OsAOS2-R | AAGGTGTCGTACCGGAGGAA | RT-qPCR |
| OsPBZ1-F | CTACTATGGCATGCTCAAGAT | RT-qPCR |
| OsPBZ1-R | ATAGAAAGGCACATAAACACAA | RT-qPCR |
| OsKS4-F | TCGCATTGCGTGTGCAA | RT-qPCR |
| OsKS4-R | TTGGAACTTCCGACATCGAAA | RT-qPCR |
| OsNAC4-F | TCCTGCCACCATTCTGAGATG | RT-qPCR |
| OsNAC4-R | TTGCAGAATCATGCTTGCCAG | RT-qPCR |
| OsPR1a-F | TGCTATGCTACGTGTTTATG | RT-qPCR |
| OsPR1a-R | AAATACGGCTGACAGTACAG | RT-qPCR |
| OsPR10b-P-F | AACACGTGTGGTGGCACGTG | RT-qPCR |
| OsPR10b-P-R | TCATCTTGAGCATGCCGAAG | RT-qPCR |
| OsMAS1-F | AAATGATTTGGGACCAGTCG | RT-qPCR |
| OsMAS1-R | GATGGAATGTCCTCGCAAAC | RT-qPCR |
| OsWRKY71-F | GCATCCGCGAGGAGTGCAAG | RT-qPCR |
| OsWRKY71-R | CCTACTTCAGATGCTCATTTGCTC | RT-qPCR |
| OsWRKY45-F | AATCGTCCGGGAATTCGGTG | RT-qPCR |
| OsWRKY45-R | GAAGTAGGCCTTTGGGTGCT | RT-qPCR |
| OsWRKY67-F | AGCGAGGAGAAGATGAGGGC | RT-qPCR |
| OsWRKY67-R | CGTTCTTGGATTTGGGCTGTTC | RT-qPCR |
| OsWRKY24-F  OsWRKY24-R | CATGTCGGAGCATTCCTTC  GGAGCCAACTCTGATCTCGT | RT-qPCR  RT-qPCR |
| OsAGO1a-F | TTGGTCTCGCAATGCTGTCT | RT-qPCR |
| OsAGO1a-R | TCGTTGGCCTATCACTCACA | RT-qPCR |
| OsAGO1b-F | GTCCAGAAGCGGCATCACAC | RT-qPCR |
| OsAGO1b-R | TAATGAGCAGGACGGCTTGTTC | RT-qPCR |
| OsAGO1c-F | TAGCCATGCTGGCATTAAGG | RT-qPCR |
| OsAGO1c-R | GAAGGCAGCCAGATGAGCATAA | RT-qPCR |
| OsAGO1d-F | CGCCACCACACGAGATTATT | RT-qPCR |
| OsAGO1d-R | GCATGGCTGCACAAGAAGAA | RT-qPCR |
| OsCERK1-F | ACGTCTACGCCTTTGGTGTT | RT-qPCR |
| OsCERK1-R | CCTTTGCGAGTTGTGTCAGC | RT-qPCR |
| OsCEBiP-F  OsCEBiP-R | AACGCTGAAGCTTGGTGAGA  ACATGGACCTAGCGAACTGC | RT-qPCR  RT-qPCR |
| OsAOS3-F  OsAOS3-R  OsEBP89-F  OsEBP89-R | ATGGTGGTCGAGAGCCACGA  CTGCTTGTTCCCCTCCGATG  GCGGCAAAGTGCAAGATCAA  CCATGTTTCTGCTGCTGCTC | RT-qPCR  RT-qPCR  RT-qPCR  RT-qPCR |
